# The Impact of SGLT-2 Inhibitors on Hydroxyl Radical Markers and Diabetic Neuropathy: A Short-Term Clinical Study

**DOI:** 10.3390/antiox14030289

**Published:** 2025-02-28

**Authors:** Ágnes Klabuzai, Viktória Bekő, Zsófia Sütő, Marcell Horváth, Zoltán Wágner, Katalin Vágási, Veronika Pfeil, Miklós Süle, György Grosz, István Wittmann, Szilárd Kun

**Affiliations:** 1 2nd Department of Medicine and Nephrology-Diabetes Centre, University of Pécs Medical School, H-7624 Pécs, Hungary; klabuzai.agnes@pte.hu (Á.K.); beko.viktoria@pte.hu (V.B.); suto.zsofia@pte.hu (Z.S.); marcell.horvath@pte.hu (M.H.); kun.szilard@pte.hu (S.K.); 2 Szigetvár Hospital Department of Neurology, Diabetology, University of Pécs Clinical Center, H-7900 Szigetvár, Hungary; 3 Unified Healthcare Institutions of the City of Pécs, H-7633 Pécs, Hungary; drwagner.zoltan@eeipecs.hu (Z.W.); vagasikati@gmail.com (K.V.); pfeilvera@gmail.com (V.P.); 4 MSB-MET Ltd., H-8230 Balatonfüred, Hungary; miklos.sule@msbmet.com (M.S.); ggrosz@msbmet.com (G.G.)

**Keywords:** diabetic kidney disease, diabetic neuropathy, hydroxyl free radical, meta-tyrosine, ortho-tyrosine, platelet, red blood cell, SGLT-2 inhibitor

## Abstract

Beyond their metabolic effect, sodium–glucose cotransporter-2 (SGLT-2) inhibitors reduce the risk of heart failure and have cardiovascular and nephroprotective effects, yet their exact mechanism of action remains unclear. This prospective study included 40 patients with type 2 diabetes whose physician initiated SGLT-2 inhibitor therapy. Prior to and 4 weeks after the initiation of SGLT-2 inhibitors, in addition to routine clinical and laboratory measurements, hydroxyl free radical and neuropathic evaluations were performed. Body weight, body mass index (BMI), fasting glucose, fructosamine, and albuminuria decreased significantly, whereas red blood cell (RBC) count, hemoglobin, hematocrit, mean corpuscular volume (MCV), and platelet count increased significantly. Urinary o-tyrosine/p-tyrosine and (m-tyrosine+o-tyrosine)/p-tyrosine ratios were significantly reduced, suggesting diminished hydroxyl free radical production. Patients with neuropathy, identified by abnormal baseline current perception threshold (CPT) values, showed significant improvements. Significant correlations between RBCs, platelet parameters, albuminuria, and hydroxyl free radical markers disappeared after SGLT-2 treatment and changes in hydroxyl free radical markers correlated positively with CPT changes. Our results suggest that short-term SGLT-2 inhibition recalibrates metabolic, hematologic, renal, and neuropathic endpoints simultaneously, presumably through attenuating abnormal ortho- and meta-tyrosine incorporation into signaling proteins. Further studies are required to confirm long-term durability and examine whether additional strategies, such as supplementation of the physiological p-tyrosine, could amplify these benefits.

## 1. Introduction

Distal symmetric polyneuropathy (DSPN) is one of the most common microvascular complications of type 2 diabetes mellitus (T2D), affecting approximately 50% of patients over the course of their disease [1]. This complex disorder involves both metabolic and vascular insults leading to nerve fiber damage, with hyperglycemia-driven oxidative stress, reduced nerve blood flow, and chronic subclinical inflammation contributing to its pathogenesis [2,3]. The presence of DSPN impairs quality of life and is associated with increased mortality [4].

Despite achieving optimal glycemic control, diabetic neuropathy frequently progresses due to multifactorial influences, including chronic oxidative stress and microvascular injury [5]. Traditional therapies have focused on symptomatic relief, such as pain management with anticonvulsants or antidepressants, and on efforts to slow disease progression by targeting hyperglycemia. Consequently, new interventions addressing additional pathogenic factors, including oxidative stress, could offer improved clinical outcomes in DSPN. It must be pointed out that oxidative stress plays a significant role in the etiology of diabetic neuropathy [6].

Under physiological conditions, phenylalanine is hydroxylated enzymatically at the para position, producing para-tyrosine. However, under conditions of increased oxidative stress, for instance, during a hydroxyl radical (OH) attack, phenylalanine can be mis-hydroxylated at the ortho- and meta-positions, giving rise to ortho-tyrosine (o-Tyr) and meta-tyrosine (m-Tyr) [5,7,8]. These atypical tyrosine isomers are typically negligible in healthy states; thus, their elevated levels or increased abnormal ratios serve as sensitive biomarkers of hydroxyl-radical-mediated oxidative damage, which may be particularly relevant in chronic diseases associated with persistent oxidative stress [5,9]. Given these oxidative stress pathways, alpha-lipoic acid (ALA) has been explored as an antioxidant therapy that primarily targets free radical production.

ALA has been utilized as a parallel or adjunctive approach to existing glycemic management strategies, with short-term trials showing significant improvements in nerve conduction velocity and neuropathic symptoms [10,11]. At the same time, ALA therapy, as a systemic antioxidant treatment, may have a beneficial effect on the cardiovascular hard endpoints, as evidenced earlier [12].

While ALA therapy underscores the importance of tackling oxidative mechanisms, sodium–glucose cotransporter-2 (SGLT-2) inhibitors are currently under investigation for potentially broader protective effects [13,14,15].

SGLT-2 inhibitors exert their primary effect in the proximal tubule of the kidney by blocking the reabsorption of glucose and sodium, resulting in insulin-independent glycemic control through enhanced urinary glucose excretion. This process induces mild osmotic diuresis and natriuresis, thereby reducing blood volume and leading to clinically relevant decreases in blood pressure, typically in the range of 2–5 mmHg [16]. Additionally, SGLT-2 inhibitors have been shown to mitigate oxidative stress and inflammation, i.e., mechanisms that may underlie the cardiovascular and renal protective effects observed [17]. Clinical trials demonstrated a clear benefit on heart failure and renal endpoints in both people with and without diabetes [18,19] while their effect on atherosclerotic endpoints is less clear and supported mainly by observational data [20].

SGLT-2 inhibitors may be associated with adverse effects, including an increased risk of diabetic ketoacidosis (DKA), genital infections, dehydration, and, in rare cases, Fournier’s gangrene [21].

Animal experiments have confirmed improved nerve conduction deficits and the prevention of intraepidermal nerve fiber loss with SGLT-2 inhibition in diabetic rat models [22,23], presumably through reduced glycemic variability, improved microvascular function, and the modulation of hydroxyl radical pathways [7,8,9].

Large cardiovascular trials (e.g., EMPA-REG, CANVAS, DECLARE) have primarily focused on cardiovascular endpoints rather than neuropathy-specific metrics [24].

In a post hoc analysis of EMPA-REG OUTCOME, changes in hematocrit alone were estimated to account for approximately 52% of empagliflozin’s cardiovascular benefit [24]. Although other major SGLT-2 inhibitor trials (DECLARE–TIMI 58, CANVAS, EMPA-KIDNEY) did not provide a similar breakdown percentage, they each reported hematocrit elevations that may reflect a possible erythropoietin-like effect—an effect further highlighted in DAPA-CKD, where increased hematocrit was thought to be a key factor in the nephroprotective benefits of the drug [17,25]. Studies suggest a variety of changes in intermediate markers (HbA1c, ketone elevation, and insulin levels decrease with anti-inflammatory effects and reductions in IL-6) in the background of the beneficial effects of SGLT-2 inhibitors. A caloric restriction-like effect is thought to be one of the most fundamental triggers [26,27].

The post hoc analysis completed for the CREDENCE study [28] found no significant difference in the number of neuropathic events between the canagliflozin-treated group and the placebo group. However, the CREDENCE trial did not include a dedicated neuropathy-specific endpoint and, in fact, the authors themselves emphasize that assessing the impact of SGLT-2 inhibitors on neuropathy requires a longer-term study with dedicated neuropathic endpoints to clarify the actual effects.

On the other hand, a small, mechanistic study proved a temporary elevation of the erythropoietin serum level using canagliflozin, which was present in the first 4 weeks of SGLT-2 therapy only, while the increase in hematocrit was prolonged for the entire 12-week-long treatment period [29]. Although there are other studies either suggesting a longer duration of erythropoietin elevation via empagliflozin treatment, even for 12 weeks [30], or no increase in elevation at 24 weeks following treatment with dapagliflozin [31], both trials verified a retained increase in RBC parameters for the whole treatment period using the SGLT-2 inhibitors.

However, to this date, few studies have systematically evaluated the short-term neuroprotective impact of SGLT-2 inhibitors alongside changes in oxidative stress markers and RBC parameters in a single cohort.

Notably, a comprehensive systematic review and meta-analysis by Kandeel [13], involving six human studies and a total of 5312 diabetic patients, demonstrated that SGLT-2 inhibitors moderately improved diabetic peripheral neuropathy symptoms, including improved sensory and motor nerve conduction velocities and potential autonomic stabilization.

In support of these findings, Ishibashi et al. [14] reported that three years of SGLT-2 inhibitor therapy in patients with modestly controlled type 2 diabetes significantly improved neuropathy outcomes and decelerated nephropathy progression.

In addition, recent perspectives emphasize that early intervention and addressing multiple metabolic factors may stabilize or even reverse nerve damage in DSPN [32] and short-term clinical evidence shows that empagliflozin can improve electrophysiological parameters, reduce oxidative stress markers, and moderate neuropathic symptoms in type 2 diabetic patients [15].

These findings point to mechanisms extending beyond glycemic control, encompassing RBC formation, changes in hydroxyl radical metabolites (such as ortho- and meta-tyrosine), and renal hemodynamics [5,7,33].

Given these encouraging data, we tested a 4-week intervention to capture immediate RBC and hydroxyl radical changes as well as early neuropathic shifts.

Taking the limited efficacy of existing treatments into account, these potential protective effects are particularly salient in the context of diabetic neuropathy’s complexity and, by directly or indirectly reducing oxidative stress and influencing pathways that underlie nerve damage, SGLT-2 inhibitors could herald a paradigm shift in the management of this challenging complication [2,6].

In light of this, our study focuses on evaluating the short-term effects of SGLT-2 inhibitor (dapagliflozin or empagliflozin) therapy on diabetic neuropathy-related measures, hydroxyl radical markers, abnormal albuminuria, and RBC parameters in patients with type 2 diabetes mellitus. Specifically, we aim to determine whether a four-week SGLT-2 inhibitor therapy could produce measurable changes in neuropathic function among T2D patients with early or mild abnormalities and to review how shifts in hydroxyl radical markers relate to these clinical and laboratory findings.

## 2. Materials and Methods

### 2.1. Study Population, Inclusion/Exclusion Criteria

Eligible participants included those whose treating physician decided to initiate SGLT-2 inhibitor therapy (empagliflozin or dapagliflozin), in addition to their existing antidiabetic regimen, based on standard clinical indications. In this study, SGLT-2 inhibitor therapy was initiated, exclusively aiming to improve glycemic control. In Hungary, the lower margin for starting SGLT-2 inhibitor therapy after metformin treatment for lowering glucose is 7.0%. Patients with severe peripheral arterial disease, a history of foot ulceration, and recurrent genital infections were also excluded. T2D patients with neuropathies of non-diabetic etiology (i.e., hereditary, drug- or toxin-induced neuropathy, untreated hypothyroidism, folate deficiency (below 3.9 µg/L), vitamin B12 deficiency (below 200 ng/L)), advanced hepatic or renal disease not meeting the inclusion criteria, autoimmune disease, malignancy, and/or regular excessive alcohol consumption (defined as more than two units per day in men and more than one unit per day in women, with one unit being equivalent to 15 g of pure alcohol) were also excluded.

**Figure 1:**
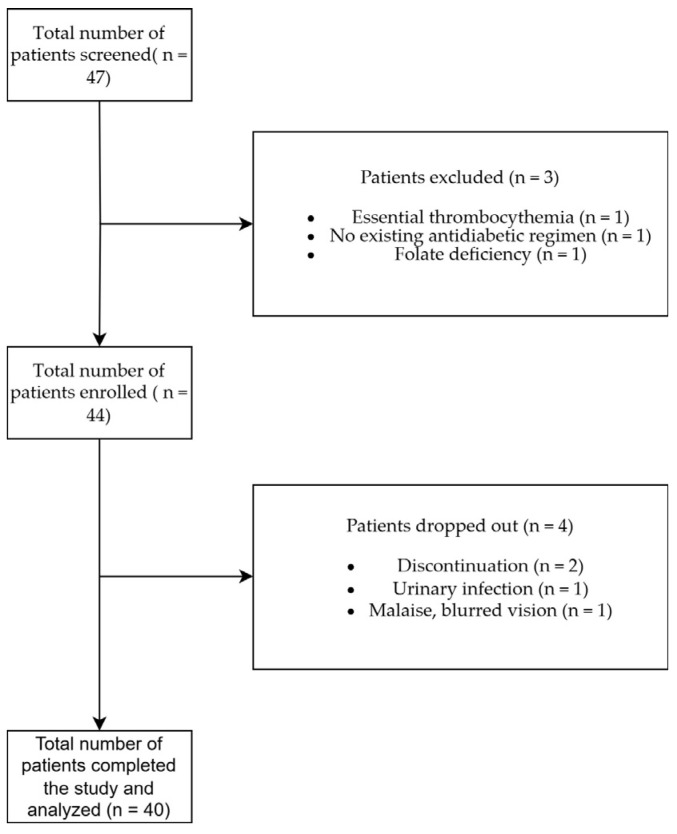
STROBE diagram of patient screening, enrollment, and study completion.

Figure 1 shows that a total of 47 patients were screened, of whom 3 were initially excluded due to essential thrombocythemia (*n* = 1), the absence of an existing antidiabetic regimen (*n* = 1), and folate deficiency (*n* = 1), resulting in a total of 44 adult T2D patients being enrolled. As this study progressed, an additional patient was excluded due to a genitourinary infection, and another one due to malaise and blurred vision, while two additional patients decided to discontinue participation. No other adverse effects of clinical significance were observed.

This study included T2D patients aged 18 to 75 years with 7% < HbA1c < 10% and eGFR > 30 mL/min/1.73 m^2^. Out of the 40 patients, none reported painful neuropathy (DN4 questionnaire), 9 exhibited neuropathic sensory symptoms (NTSS questionnaire), and 9 showed loss of protective sensation (monofilament test). According to the Neuropathy Disability Score (NDS), 14 patients had mild neuropathy, 16 had moderate neuropathy, and 4 had severe neuropathy, indicating that most patients had preexisting asymptomatic neuropathy.

### 2.2. Study Design and Objectives

This prospective, open-label, single-arm clinical study aimed to investigate the short-term effects of sodium-glucose cotransporter-2 (SGLT-2) inhibitors on distal symmetric diabetic polyneuropathy, hydroxyl radical markers, and related hematological and renal parameters in patients with type 2 diabetes mellitus (T2D). The rationale was based on the putative mechanism whereby, beyond their glucose-lowering effect, SGLT-2 inhibitors may influence hydroxyl radical marker formation and thereby improve microvascular complications. This includes the potential beneficial effects on red blood cell (RBC) parameters, abnormal albuminuria, and early-stage diabetic neuropathy. The primary hypothesis was that short-term SGLT-2 inhibitor therapy would mitigate hydroxyl free radical pathways, specifically tyrosine metabolite ratios accompanied by improved neuropathic measurements and RBC-related indices.

### 2.3. Intervention and Study Protocol

All participants underwent a baseline (Week 0) assessment followed by a follow-up evaluation after 4 weeks of SGLT-2 inhibitor therapy. The dose and choice of SGLT-2 inhibitor were determined by the clinical team based on standard treatment indications. No additional interventions were mandated by this study.

### 2.4. Data Collection and Questionnaires

Participants completed lifestyle and medication questionnaires at baseline, documenting diet, exercise, smoking and alcohol consumption habits, concomitant medications, and comorbidities. The Neuropathic Total Symptom Score (NTSS) and DN4 questionnaires were administered to evaluate subjective neuropathic complaints.

### 2.5. Neuropathic Assessments

DSPN is one of the most common complications in patients with type 2 diabetes (T2D), even in those with well-controlled glycemia. DSPN can affect either small or large nerve fibers or a combination of the two in a length-dependent manner in a “stocking-glove” distribution [32]. The neuropathy evaluation was performed at the Neuropathy Center of the 2nd Department of Medicine and Nephrology-Diabetes Center, University of Pécs Medical School.

Functional and symptomatic clinical examinations were conducted for distal symmetric polyneuropathy (DSPN):

1. Deep tendon reflex testing and 128 Hz calibrated tuning fork (vibration) for large-fiber dysfunction;
2. Pinprick (pain) and tiptherm (temperature) tests for small-fiber dysfunction;
3. A 10 g monofilament (protective sensation) evaluation for the risk of developing foot ulcers [32];
4. DN4 questionnaire for painful neuropathy assessment;
5. NTSS-6 questionnaire for the evaluation of sensory symptoms;
6. NDS calculation for rating the severity of DSPN;
7. Quantitative sensory testing was conducted, using the CA-12 software-based system (MSB-MET Ltd., Balatonfüred, Hungary), to measure the current perception thresholds (CPTs) at three frequencies (2000 Hz, 250 Hz, and 5 Hz) on both the median and peroneal nerve, providing insight into large myelinated (2000 Hz Aβ), medium-size myelinated (250 Hz Aδ), and unmyelinated (5 Hz C) small-fiber functions. CPT values below the normal range indicate hyperesthesia while values above the normal range indicate hypesthesia. CPT is a suitable instrument for the assessment of DSPN and is able to detect even slight neuropathic abnormalities compared to NCS tests, especially in the case of small fibers. Therefore, we primarily relied on the analysis of deviations in CPT values during the design of our research [34]. This parameter was determined as the primary endpoint.

### 2.6. Laboratory Measurements

Blood and urine samples were collected at baseline and at Week 4. No additional venipuncture was required beyond routine sampling; approximately 10 mL of blood was obtained for research purposes.

#### 2.6.1. Routine Parameters

The HbA1c was measured at Week 0. Serum fructosamine, fasting glucose, and serum insulin were measured at both Week 0 and Week 4. Serum creatinine and the estimated glomerular filtration rate (eGFR) were assessed at Week 0 and Week 4. Serum bilirubin, AST, ALT, GGT, ALP, LDH, INR, serum total protein, albumin, total cholesterol, HDL, LDL, uric acid serum iron, transferrin, transferrin saturation, and ferritin were measured at Week 0, whereas triglycerides, serum sodium, potassium, the erythrocyte sedimentation rate (ESR), C-reactive protein (CRP), and urinary albumin and creatinine were assessed at both Week 0 and Week 4.

#### 2.6.2. Hydroxyl Free Radical Markers

To explore the hydroxyl free radical pathway and its link to neuropathy, RBC-formation, and renal parameters, markers were measured at the baseline and at Week 4. Serum and urinary phenylalanine (Phe), para-tyrosine (p-Tyr), meta-tyrosine (m-Tyr), and ortho-tyrosine (o-Tyr) were quantified via high-performance liquid chromatography (HPLC). In addition to the direct concentrations of these metabolites, the following ratios were calculated to characterize changes in the oxidative milieu under SGLT-2 inhibitor therapy (p-Tyr/Phe, m-Tyr/Phe, o-Tyr/Phe, (m-Tyr+o-Tyr)/Phe, m-Tyr/p-Tyr, o-Tyr/p-Tyr, (m-Tyr+o-Tyr)/p-Tyr, m-Tyr/krea, p-Tyr/krea, o-Tyr/krea) as well as any corresponding serum and urinary values. This comprehensive panel of hydroxylation products and derived ratios was intended to provide a detailed assessment of hydroxyl free radical activity and its potential influence on diabetic complications.

#### 2.6.3. Additional Calculations

The Homeostasis Model Assessment of Insulin Resistance (HOMA_IR_) was calculated to assess insulin sensitivity. Changes in the urinary albumin/creatinine ratio (ACR) and correlations with current perception threshold (CPT) parameters were also evaluated throughout this study.

The Neuropathy Disability Score (NDS) was calculated using a standardized clinical examination approach [35]. This assessment evaluates ankle reflexes, vibration sensation, pin-prick sensation, and temperature sensation at the big toe. The maximum total score is 10, and the scores are categorized as follows:

- 3–5 points: mild neuropathy;
- 6–8 points: moderate neuropathy;
- 9–10 points: severe neuropathy.

#### 2.6.4. High-Performance Liquid Chromatography (HPLC) Analyses

Venous blood was taken from patients and collected into native tubes during routine blood sampling. Serum was extracted via centrifugation and was stored at −80 °C pending further examination. Thereafter, 125 µL of trichloro-acetic acid (TCA) was added to 500 µL of serum and samples were taken and then incubated on ice for 30 min. Afterward, the precipitate was separated via centrifugation and the supernatant was filtered using a 0.2 μm syringe filter (Millipore Inc., Darmstadt, Germany). The supernatant was stored at −80 °C until undergoing processing or was immediately analyzed via HPLC.

A HPLC analysis was carried out using a Shimadzu Class LC-10 ADVP HPLC device (Shimadzu Manufacturing Inc., Canby, OR, USA). An amount of 20 μL of the filtrate was injected onto a Licrospher-C18 silica column (Merck, Darmstadt, Germany). Different amino acids and isomers, namely, phenylalanine (Phe), para-tyrosine (p-Tyr), meta-tyrosine (m-Tyr), and ortho-tyrosine (o-Tyr), were detected using their autofluorescence. Additionally, p-, m-, and o-Tyr were measured at 275 nm excitation and 305 nm emission while Phe was evaluated at 258 nm excitation and 288 nm emission using a Shimadzu RF-10AXL fluorescent detector (Shimadzu Manufacturing Inc., Canby, OR, USA). An isocratic flow of the eluent containing 1% acetic acid, 1% sodium acetate, and 98% distilled water was performed. To determine the concentrations of different amino acids and isomers, external standards were used.

### 2.7. Statistical Analysis

Data were analyzed by a biostatistician using appropriate methods for paired comparisons and performing correlation and regression analyses to identify potential predictors of change in neuropathy, hydroxyl-radical-related, and metabolic parameters. Subgroup analyses reviewing patients with abnormal baseline CPT values were performed to discern whether SGLT-2 inhibitor therapy might confer greater neurological benefits in those with pre-existing neuropathic alterations. The distribution of parameters was tested using Kolmogorov–Smirnov’s normality test. Changes in parameters between baseline and Week 4 were compared using a paired sample *t*-test. Correlational analyses were performed via Pearson’s (normal distribution) or Spearman’s rho (non-normal distribution) tests. Correlations were used as explorative, secondary endpoints. Linear regression models constructed via the stepwise method were established for evaluating predictors of neuropathy, RBC parameters, and albuminuria. The Statistical Package for Social Sciences (SPSS) version 28 (IBM, Armonk, New York, NY, USA) was used for statistical analyses; a ‘*p*’ value below 0.05 was considered statistically significant.

In summary, the methods were designed to investigate whether short-term SGLT-2 inhibitor therapy, performed by reducing hydroxyl free radical generation and improving RBC indices and abnormal albuminuria, could also yield measurable improvements in diabetic neuropathic function and related hydroxyl free radicals.

## 3. Results

Table 1 presents the changes in anthropometric, hemodynamic, neuropathic, metabolic, kidney-function-related, inflammatory, hematological, and hydroxyl free radical parameters in 40 patients (19 male/21 female) undergoing 4 weeks of SGLT-2 inhibitor therapy (22 empagliflozin/18 dapagliflozin). The data are expressed as mean ± SD and *p*-values are derived from paired-sample *t*-tests comparing values at baseline (Week 0) and after treatment (Week 4).

Notably, body weight (*p* = 0.004), BMI (*p* = 0.006), fasting glucose (*p* = 0.038), fructosamine (*p* = 0.001), abnormal albuminuria (MAU) (*p* = 0.032), and standing systolic blood pressure (SBP) (*p* = 0.020) decreased significantly (Table 1). RBC count (*p* = 0.020), hemoglobin (*p* = 0.029), hematocrit (*p* = 0.002), MCV (*p* = 0.014), and platelet count (*p* = 0.022) increased significantly (Table 1). Significant reductions were detected in urinary o-Tyr/p-Tyr (*p* = 0.020) and (m-Tyr+o-Tyr)/p-Tyr (*p* = 0.024) ratios (Table 1).

Table 2 illustrates the current perception threshold (CPT) measurements obtained at baseline (Week 0) and after 4 weeks of SGLT-2 inhibitor therapy in 40 patients. CPT values, expressed as mean ± SD, were assessed at three frequencies (2000 Hz, 250 Hz, 5 Hz) for both the peroneal and median nerves, bilaterally.

No statistically significant changes were observed after 4 weeks in the results of the monofilament, pinprick, tiptherm, calibrated tuning fork, DN4, NTSS, and NDS tests (*p* > 0.05).

No statistically significant changes in CPT were noted at any frequency or nerve site following the 4-week treatment period. Both small- and large-fiber functions remained stable. These findings suggest that short-term SGLT-2 inhibition did not induce any generalized changes in CPT results in the entire T2D patient group during the observation period.

As no significant changes were found in the CPT results for the whole test population, we presume that a short-term effect may manifest in patients who had pathological CPT values (i.e., those exceeding the normal range) even at the onset. For this reason, certain sub-groups were analyzed separately.

**Figure 2:**
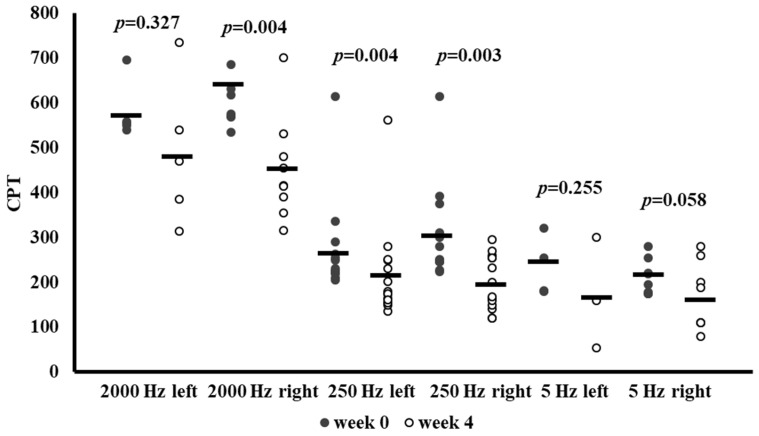
Current perception threshold (CPT) values at baseline (Week 0) and after 4 weeks of SGLT-2 inhibitor therapy in patients whose peroneal nerve CPTs were above the normal range at baseline. Individual data points are presented with mean CPT (10 μA) at 2000 Hz, 250 Hz, and 5 Hz, measured on the left and right sides and *p*-values from paired-sample *t*-tests comparing Week 0 and Week 4. Abbreviation: CPT, current perception threshold.

In the subgroup of individuals who presented above-normal baseline CPT measurements on the peroneal nerve at the frequency concerned, Figure 2 shows the effects of SGLT-2 inhibitor therapy on current perception threshold (CPT) values at Week 0 and Week 4. CPTs were recorded at three frequencies (2000 Hz, 250 Hz, 5 Hz) for both the left and right sides.

In this subgroup, a significant reduction in CPT at 2000 Hz was observed on the right side (*p* = 0.004) and at 250 Hz on both sides (left: *p* = 0.004, right: *p* = 0.003). Although CPT values also decreased at 5 Hz on both sides and at 2000 Hz on the left side, presumably due to the low sample size, these changes did not reach statistical significance. These improvements suggest that SGLT-2 inhibitor therapy may beneficially influence peroneal nerve function in patients with abnormal baseline values.

**Figure 3:**
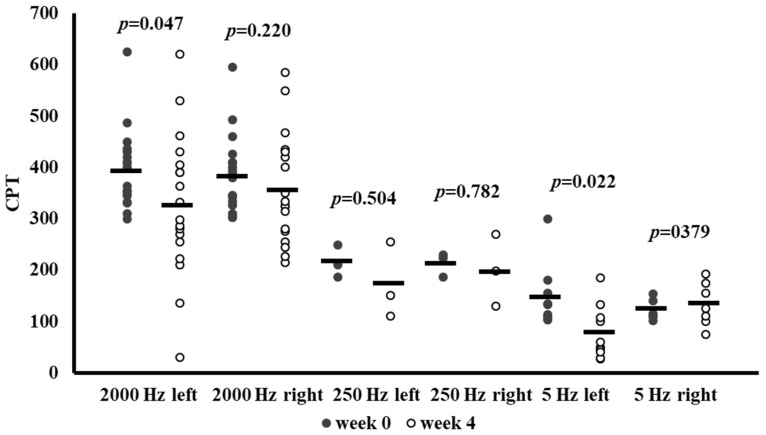
Current perception threshold (CPT) values at baseline (Week 0) and after 4 weeks of SGLT-2 inhibitor therapy in patients whose median nerve CPTs were above the normal range at baseline. Individual data points are presented with mean CPT (10 μA) at 2000 Hz, 250 Hz, and 5 Hz measured on the left and right sides, along with the *p*-values from paired-sample *t*-tests comparing Week 0 and Week 4. Abbreviation: CPT, current perception threshold.

Figure 3 illustrates CPT measurements on the median nerve at Week 0 and Week 4 in patients with above-normal baseline CPT values at the given frequency. CPT was assessed at 2000 Hz, 250 Hz, and 5 Hz on both the left and right sides.

Following 4 weeks of SGLT-2 inhibitor therapy, a significant reduction in CPT at 2000 Hz was observed on the left side (*p* = 0.047) and at 5 Hz on the left side (*p* = 0.022). The reduction in CPT at 2000 Hz on the right side did not reach statistical significance; the numeric trend aligns with the overall pattern of beneficial changes. Similarly to the results of the peroneal nerve, no significant changes were observed in the subgroups with a lower sample size (250 Hz left and right, 5 Hz right). Despite significant improvements being observed at only two of the six sites, these results also suggest that SGLT-2 inhibitors may exert beneficial effects on median nerve function in patients with initially elevated CPTs.

**Figure 4:**
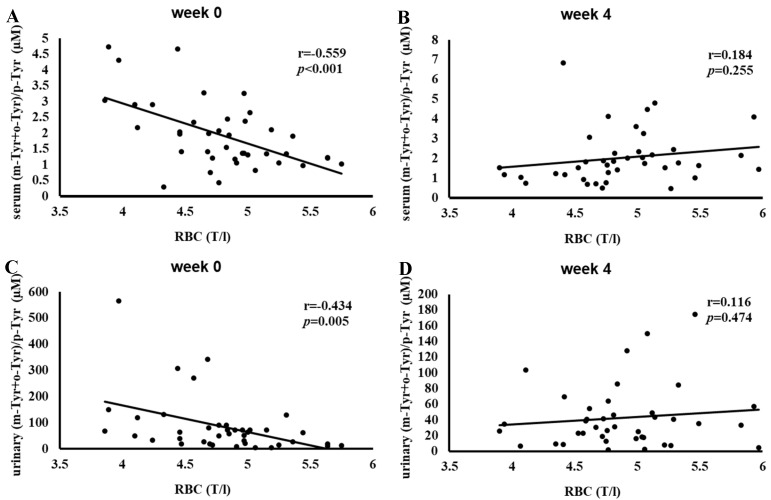
Correlation between red blood cell (RBC) count and the (m-Tyr+o-Tyr)/p-Tyr ratio in serum (panels (**A**,**B**)) and urine (panels (**C**,**D**)) at Week 0 (**A**,**C**) and Week 4 (**B**,**D**) of SGLT-2 inhibitor therapy. Each panel indicates the Pearson correlation coefficient (*r*) and corresponding *p*-value. The fitted line represents the linear regression trend. Abbreviations: RBC, red blood cell; m-Tyr, meta-tyrosine; o-Tyr, ortho-tyrosine; p-Tyr, para-tyrosine.

Figure 4 depicts the correlation between red blood cell (RBC) counts and serum and urinary (m-Tyr+o-Tyr)/p-Tyr ratios at baseline (Week 0) and after 4 weeks of SGLT-2 inhibitor therapy.

At Week 0, higher RBC counts correlated significantly with lower (m-Tyr+o-Tyr)/p-Tyr ratios in both serum (r = −0.559, *p* < 0.001) and urine (r = −0.434, *p* = 0.005), indicating an inverse relationship between RBC levels and hydroxyl free radical markers (Figure 4).

By Week 4, these correlations were no longer significant. This loss of association suggests that SGLT-2 inhibitor therapy may have altered the oxidative and metabolic conditions affecting RBC parameters, reducing the relevance of these baseline hydroxyl radical markers (Figure 4).

**Figure 5:**
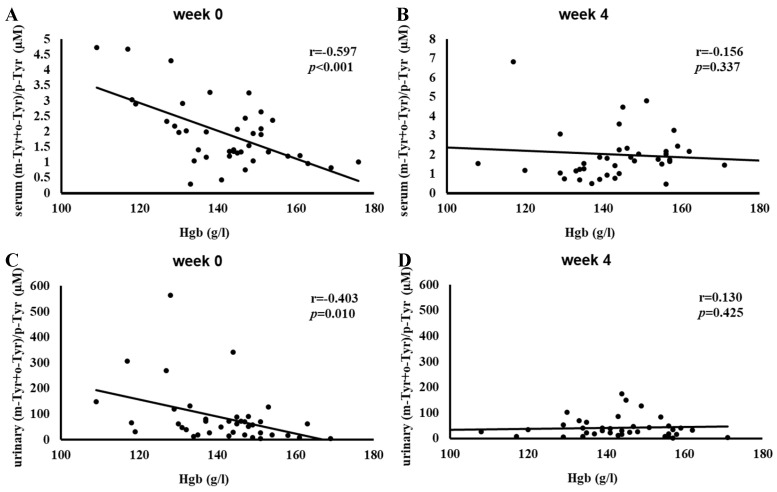
Correlation between hemoglobin (Hgb) levels and the (m-Tyr+o-Tyr)/p-Tyr ratio in serum (panels (**A**,**B**)) and urine (panels (**C**,**D**)) at Week 0 (**A**,**C**) and Week 4 (**B**,**D**) of SGLT-2 inhibitor therapy. Each panel indicates the Pearson correlation coefficient (*r*) and corresponding *p*-value. The fitted line represents the linear regression trend. Abbreviations: Hgb, hemoglobin; m-Tyr, meta-tyrosine; o-Tyr, ortho-tyrosine; p-Tyr, para-tyrosine.

Figure 5 shows the correlation between hemoglobin (Hgb) levels and serum and urinary (m-Tyr+o-Tyr)/p-Tyr ratios at Week 0 and Week 4.

Initially, higher Hgb levels were significantly associated with lower (m-Tyr+o-Tyr)/p-Tyr ratios in serum (r = −0.597, *p* < 0.001) and urine (r = −0.403, *p* = 0.01), reflecting a strong inverse relationship at baseline.

After 4 weeks of SGLT-2 inhibitor therapy, these associations were no longer significant. The disappearance of these baseline correlations indicates that treatment may have changed the oxidative environment linked to Hgb levels.

**Figure 6:**
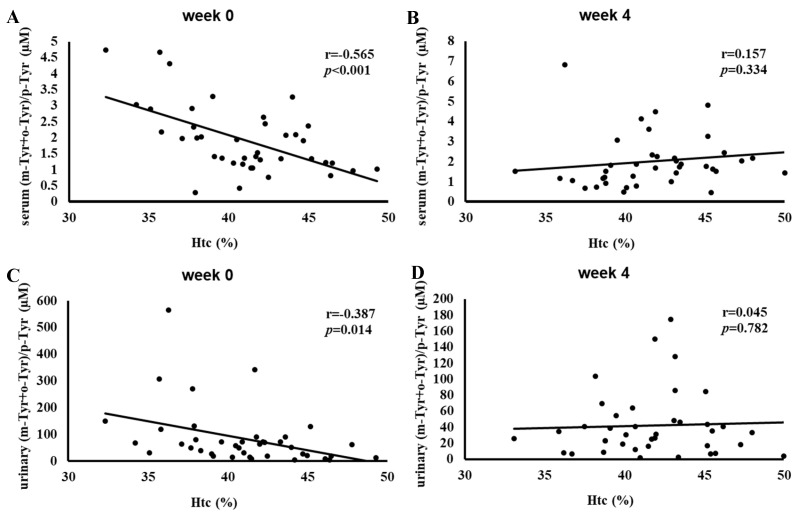
Correlation between hematocrit (Htc) and the (m-Tyr+o-Tyr)/p-Tyr ratio in serum (panels (**A**,**B**)) and urine (panels (**C**,**D**)) at Week 0 (**A**,**C**) and Week 4 (**B**,**D**) of SGLT-2 inhibitor therapy. Each panel indicates the Pearson correlation coefficient (*r*) and corresponding *p*-value. The fitted line represents the linear regression trend. Abbreviations: Htc, hematocrit; m-Tyr, meta-tyrosine; o-Tyr, ortho-tyrosine; p-Tyr, para-tyrosine.

Figure 6 illustrates the correlation between hematocrit (Htc) levels and serum and urinary (m-Tyr+o-Tyr)/p-Tyr ratios at Week 0 and Week 4. At baseline, higher Htc levels correlated negatively with (m-Tyr+o-Tyr)/p-Tyr ratios in serum (r = −0.565, *p* < 0.001) and urine (r = −0.387, *p* = 0.014).

By Week 4, these significant correlations were lost. This shift once again suggests that SGLT-2 inhibitor therapy influenced the oxidative and metabolic factors driving these relationships, contributing to a more balanced redox environment.

Figure 4, Figure 5 and Figure 6 show a similar pattern: before SGLT-2 inhibitor therapy, there were strong inverse relationships between red-blood-cell-related parameters (RBC count, hemoglobin, and hematocrit) and hydroxyl free radical markers ((m-Tyr+o-Tyr)/p-Tyr ratios) in both serum and urine. At baseline, higher RBC parameters corresponded to lower levels of these hydroxyl free radical parameters, indicating a clear link between the body’s red blood cell status and its oxidative state.

After 4 weeks of SGLT-2 inhibitor therapy, these significant correlations ceased to exist. This loss of association suggests that the treatment modified the underlying oxidative and metabolic conditions that previously tied RBC-related factors to this marker of hydroxyl free radicals. In essence, while RBC count, hemoglobin, and hematocrit served as meaningful proxies for redox balance at the start of this study, short-term SGLT-2 inhibition altered the biochemical environment to such an extent that these relationships no longer exist.

As far as platelets are concerned, a positive correlation was proved between the serum (m-Tyr+o-Tyr)/p-Tyr ratio and the platelet count (R = 0.320, *p* = 0.044; Figure S1), an association which disappeared after 4 weeks of SGLT-2 inhibitor therapy (R = 0.301, *p* = 0.059; Figure S1).

Similarly, before the treatment, a significant positive correlation was found between albuminuria and urinary (m-Tyr+o-Tyr)/p-Tyr (R = 0.446, *p* = 0.006; Figure S2) but 4 weeks of SGLT-2 inhibitor therapy eliminated this association (R = −0.050, *p* = 0.775; Figure S2).

**Figure 7:**
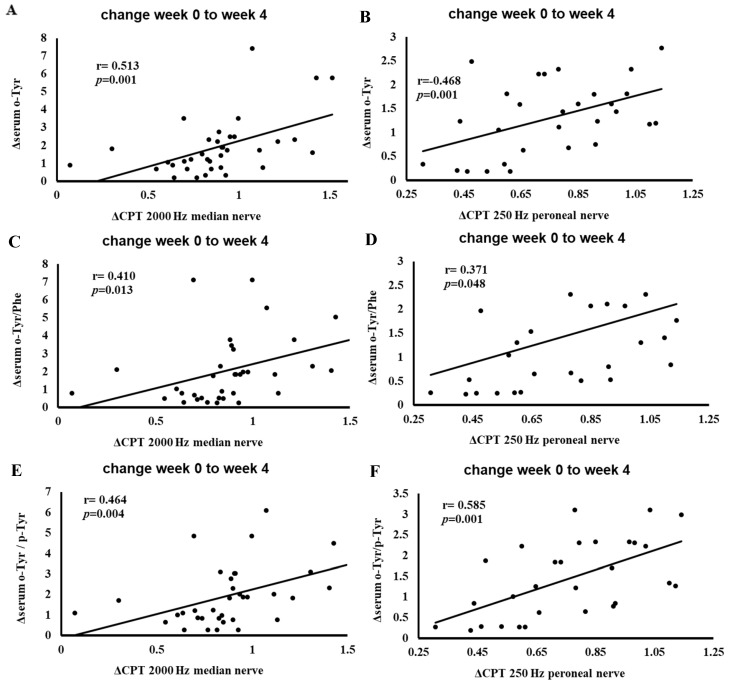
Correlation between changes (Week 0 to Week 4) in serum o-Tyr, o-Tyr/Phe, and o-Tyr/p-Tyr and changes (Week 0 to Week 4) in CPT results at the 2000 Hz median nerve (panels (**A**,**C**,**E**)) and 250 Hz peroneal nerve (panels (**B**,**D**,**F**)). Analyses include patients whose baseline (Week 0) CPT values were above the normal range for the given nerve and frequency (*n* = 25). Each panel indicates the Pearson correlation coefficient (r) and corresponding *p*-value. The fitted line represents the linear regression trend. Abbreviations: CPT, current perception threshold; o-Tyr, ortho-tyrosine; Phe, phenylalanine; p-Tyr, para-tyrosine.

Figure 7 depicts the correlations between changes in serum hydroxyl free radical markers (o-Tyr, o-Tyr/Phe, o-Tyr/p-Tyr) and changes in CPT results at 2000 Hz on the median and 250 Hz on the peroneal nerves (frequencies with the most number of cases on the nerve site concerned) from Week 0 to Week 4.

Significant positive correlations emerged, indicating that patients who experienced favorable shifts in hydroxyl free radical parameters also demonstrated improvements in CPT measures. These findings suggest a link between oxidative adjustments due to SGLT-2 inhibitor therapy and enhanced nerve function, particularly in patients presenting abnormal baseline CPT values.

Table 3 summarizes the predictors of peripheral neuropathy (at various frequencies), red blood cell parameters (Hgb and Htc), and abnormal albuminuria (MAU) at baseline (Week 0), prior to the initiation of SGLT-2 inhibitor therapy.

At Week 0, several hydroxyl free radical markers and other metabolic, hemodynamic, and anthropometric factors significantly predicted neuropathy severity, indices of RBCs, and MAU. By Week 4, all these baseline predictive relationships disappeared, suggesting that SGLT-2 inhibitor therapy modulated the underlying oxidative and metabolic conditions. This shift aligns with the changes observed in correlation patterns, supporting the concept that SGLT-2 inhibition can reshape the oxidative–metabolic landscape influencing neuropathy, RBC parameters, and renal function.

## 4. Discussion

In this study, we have demonstrated that our findings suggest that a short-term (4-week) therapy with SGLT-2 inhibitors in T2D patients may exert multifaceted benefits on metabolic, renal, hematological, and neuropathic endpoints parallel with the changes in the hydroxyl free radical exposure.

Due to the lack of a control group, possible cofounders, e.g., lifestyle factors, such as diet changes or the fluctuation in physical activity, cannot be excluded but body weight, BMI, fasting glucose, and fructosamine decreased significantly, indicating that our cohort achieved better glycemic control and experienced modest weight reduction, findings that are consistent with the known metabolic effects of SGLT-2 inhibitors [16,17].

The major cardiovascular outcome trials (e.g., EMPA-REG, CANVAS, DECLARE) primarily focused on cardiovascular endpoints and did not include specific analyses targeting neuropathy. The post hoc analysis related to the CREDENCE study [28] concluded that the incidence of neuropathic events did not decrease significantly in the canagliflozin group compared to the placebo-treated group. However, this may be partly attributed to the absence of a dedicated neuropathy-specific endpoint in this study. Therefore, the authors similarly advocate a longer-term SGLT-2 trial specifically focusing on neuropathy.

In the meantime, new evidence has emerged in the past few years suggesting the fact that in addition to cardiometabolic effects, SGLT-2 inhibitors may have neuroprotective benefits in terms of diabetic neuropathy. For instance, a meta-analysis [13] has revealed that SGLT-2 inhibitors can mildly improve certain endpoints of DSPN and nerve conduction velocities. In addition, based on the results of a 3-year longitudinal study, SGLT-2 inhibitor treatment significantly improved certain neuropathic symptoms [14]. In line with this, a short-term randomized trial [15] revealed that even empagliflozin administration as short as 3 months could result in significant improvements in electrophysiological studies and oxidative stress marker values in patients with type 2 diabetes.

Based on these findings, our results now indicate that even a brief, 4-week SGLT-2 inhibitor therapy may induce measurable improvements in hydroxyl-free-radical-driven amino acid modifications while concurrently improving current perception threshold (CPT) values in patients with type 2 diabetes who initially exhibited abnormal (above-range) CPT values.

Although no significant CPT changes were observed during the four-week study period in the entire study population, the subgroup-specific effects correspond with findings from the 3-month follow-up study [15], further reinforcing the hypothesis, whereby reducing oxidative stress—particularly through the mitigation of hydroxyl free radical formation—may facilitate the emergence of early neuroprotective effects, even during a relatively short treatment period in diabetic neuropathy. Therefore, it is of critical importance to further investigate this effect in a longer-term, randomized, controlled trial in the future.

Crucially, we observed a notable decoupling between red blood cell (RBC) and platelet parameters and hydroxyl-free-radical-derived markers by the end of the study period.

Notably, while lower RBC and higher platelet levels were tied to higher hydroxyl free radical production at baseline, these relationships were no longer observed after four weeks of SGLT-2 inhibitor therapy. This decoupling implies that improved metabolic and hormonal conditions brought about by SGLT-2 inhibitors may reduce the production of hydroxyl radicals [7,8], leading to improved RBC parameters, probably by mitigating hormonal resistances.

**Figure 8:**
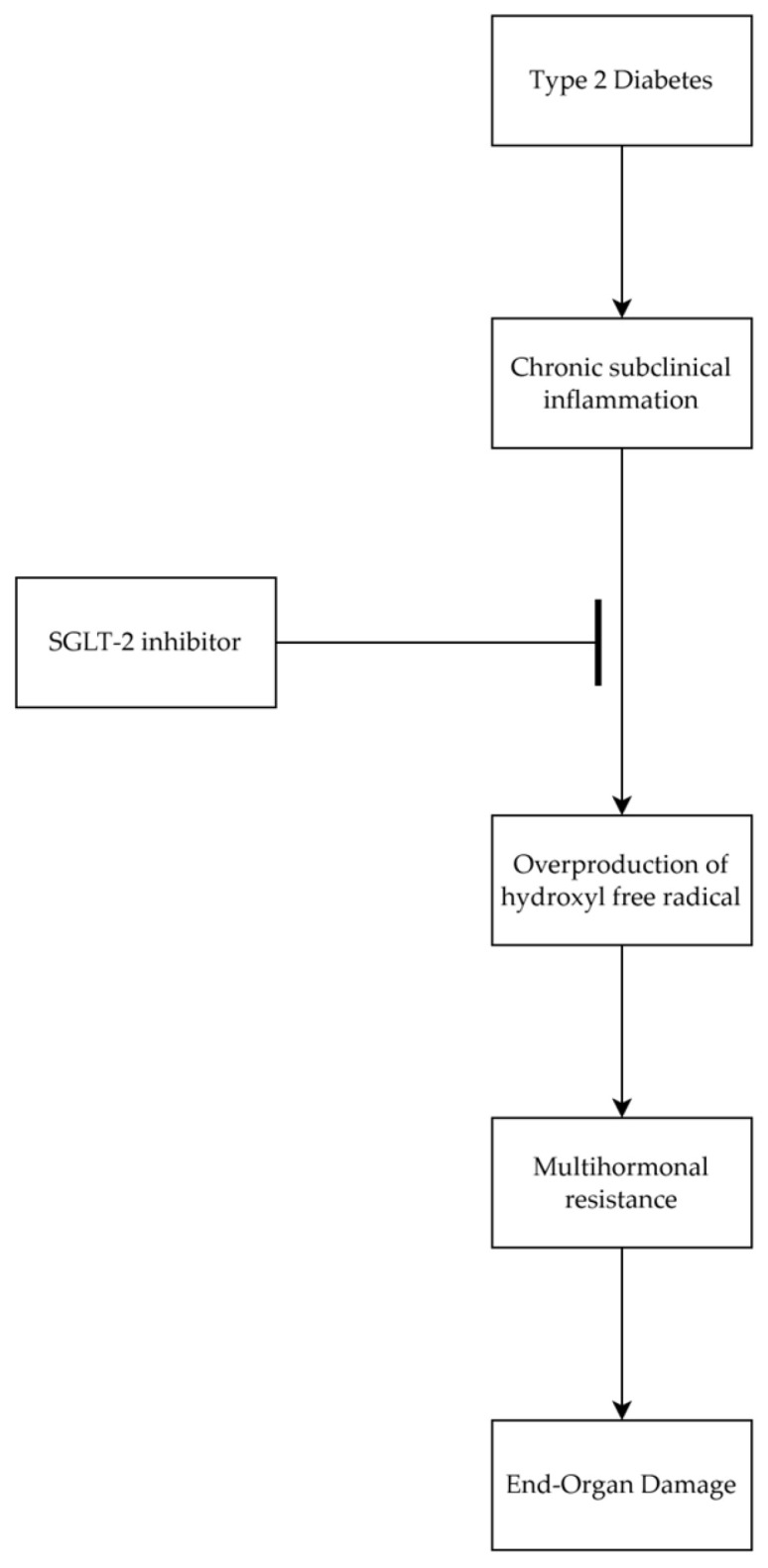
Schematic overview of our study’s proposed mechanism.

Figure 8 depicts how chronic subclinical inflammation results in excessive hydroxyl free radical production in type 2 diabetes, contributing to abnormal tyrosine isomer incorporation into key signaling proteins and subsequent multihormonal resistance. This can lead to end-organ damage (e.g., neuropathy). Our findings suggest that short-term SGLT-2 inhibitor therapy may help interrupt this pathway by attenuating hydroxyl free radical overproduction, potentially mitigating the early stages of diabetic organ complications. While the mechanism proposed offers a convincing framework, further controlled research is required to confirm these processes and to corroborate the possible therapeutic targets.

A well-known clinical phenomenon is the incidence of lower RBC and higher platelet counts in chronic oxidative stress, e.g., in metabolic syndrome [36]. We suppose the effect of multihormonal resistance to be erythropoietin, insulin, and thrombopoietin resistance in the background of these hematological abnormalities. Moreover, the antiapoptotic effects of these hormones are necessary not only for the physiological hematopoiesis but also for the defense of other organs, e.g., the kidney, heart, and neurons.

Another key finding of our study is the significant reduction in abnormal albuminuria, reflecting evidence from large-scale trials that underscore the nephroprotective properties of SGLT-2 inhibitors [17,25].

While neuropathy metrics for the entire cohort remained largely unchanged, our subgroup analysis revealed considerable improvement in current perception threshold (CPT) values among individuals presenting with abnormal baseline readings. The decrease in hydroxyl free radical markers also correlated positively with improvements in CPT (Figure 7), suggesting that the decoupling of oxidative stress may play a role in facilitating early nerve function gains in patients showing signs of neuropathic dysfunction [14], similar to the negative correlation found between MDA and sensory electrophysiological parameters in a previous study [15].

Moreover, the changes in associations between o- or m-Tyr and the number of RBC and platelets and improvement in albuminuria and neuropathy due to the SGLT-2 inhibitor therapy may also support another approach. In this sense, o- and m-Tyr could not only be markers but also makers since these abnormal tyrosines, according to our previous observations, could be incorporated into the signaling proteins leading to multihormonal resistances [5].

We proved the development of o-Tyr- and/or m-Tyr-induced insulin [9], acetylcholine [33], and erythropoietin [37] resistances, which together could lead to abnormal albuminuria and neuropathy and a lower RBC count.

In the case of o- and m-Tyr-induced erythropoietin-resistant RBC malformation, we verified a STAT- and ERK-dependent signaling defect [37], whose pathways also contribute to the thrombopoietin-enhanced platelet production together with the Akt signaling [38], for which an o- and m-Tyr-dependent defect was also demonstrated by our workgroup [5].

This study aimed to detect markers derived solely at the intracellular level, the presumed pathophysiological origin of the favorable changes that eventually affect the entire body. Further tests are required with a larger sample, the involvement of a control group, and a longer follow-up period in order to corroborate the significance of the presence of these intracellular markers in clinical outcomes. The ominous products of phenylalanine are reliable markers of hydroxyl free radical production, a process that occurs only within cells. Subsequently, these changes can also be detected at higher levels (in circulating hormones, cytokines, glycemia, and at the level of organs: red blood cell production, neuropathy, atherosclerosis, kidney damage, etc.). It is important to note that euglycemia is not necessarily associated with eumetabolism and acute hyperglycemia does not automatically indicate dysmetabolism [39]. Similarly, due to the lack of the elimination of confounding factors, the short-term changes detected in glycemic parameters during the four-week period do not necessarily reflect the overall metabolic improvements or the long-term organic protection.

## 5. Limitations

This study is a preliminary investigation conducted on a small patient population (*n* = 40) without a control group and randomization. As a result, the findings derived from this study are hypothesis-generating in nature and limited in both statistical power and generalizability. The short, 4-week study duration may not be sufficiently long to provide a reliable assessment of the long-term effects of SGLT-2 inhibitors on neuropathy, particularly given that diabetic neuropathy is typically a slowly progressive condition. Nevertheless, several parameters (body weight, BMI, fasting glucose, fructosamine, abnormal albuminuria (MAU), standing systolic blood pressure, RBC count, hemoglobin, hematocrit, MCV, platelet count, and urinary o-Tyr/p-Tyr, and (m-Tyr+o-Tyr)/p-Tyr) showed significant changes, even within this short time frame.

From a methodological perspective, another limitation is that our significant findings regarding the improvement of neuropathic status are derived from subgroup CPT measurements, whereas no significant differences were observed in other functional and symptomatic tests. Quality of life and nerve conduction velocity were not assessed. In addition, we could not eliminate all potential confounding factors during this study (e.g., physical activity, dietary changes). Moreover, despite the relatively small sample size, we conducted multiple comparisons across numerous parameters, which may have increased the risk of false-positive results due to multiple tests. Another limitation is that *p*-values were not adjusted during correlational analyses.

Although our findings suggest that even a short course of therapy may lead to measurable changes in hydroxyl free radical markers and neuropathic status in certain subgroups, randomized, controlled, mechanistic studies are essential to establish a true causal relationship. Nevertheless, this preliminary study can pave the way for further research with longer follow-up periods and larger patient cohorts, which allows a more precise mapping of the short- and long-term effects of SGLT-2 inhibitors on hydroxyl free radical formation, hormone resistance, and neuropathy.

## 6. Conclusions

Overall, these findings suggest that short-term SGLT-2 inhibition may trigger a coordinated recalibration of metabolic and redox processes, decoupling RBC-related parameters from hydroxyl radical production and enabling early neuropathic improvements in patients with pre-existing peripheral nerve dysfunction. By reducing albuminuria, increasing RBC indices and platelet count, and selectively enhancing nerve function, SGLT-2 inhibitors appear to engage multiple, interlinked pathogenic pathways of which common roots may be found in the incorporation of abnormal tyrosines into the key signaling proteins due to the overproduction of hydroxyl free radicals changing the way intracellular phosphorylation cascades and leading to multihormonal resistance and, as a consequence, to multiorgan defects. These data underscore the need to look beyond simple glycemic targets when evaluating the therapeutic value of SGLT-2 inhibitors, highlighting novel opportunities for optimizing interventions aimed at mitigating diabetic complications. Nonetheless, further research is warranted to confirm the durability of these short-term changes, clarify causality, and investigate whether additional strategies to attenuate the effect of hydroxyl free radicals could enhance the overall benefits of SGLT-2 inhibitors for patients with diabetic complications.

## Figures and Tables

**Table 1:** The effect of SGLT-2 inhibitor therapy on clinical and hydroxyl radical parameters.

	Week 0	Week 4	*p*
*n*	40		
Age (year)	61 ± 9		
Gender (male [%])	19 [47.5]		
Diabetes duration (year)	8.3 ± 8.0		
Dapagliflozin/empagliflozin [%]	18/22 [45/55]		
Body weight (kg)	92.52 ± 16.78	90.4 ± 15.01	0.004
BMI (kg/m^2^)	32.6 ± 4.45	31.94 ± 4.19	0.006
Hemodynamic and neuropathic parameters			
RR distance in SR (ms)	808.16 ± 94.19	832.05 ± 82.37	0.054
SBP supine (mmHg)	143 ± 16.85	138.05 ± 19.78	0.106
DBP supine (mmHg)	87.78 ± 10.43	85.73 ± 9.14	0.241
HR supine (bpm)	77.7 ± 10.55	75.1 ± 8.57	0.091
SBP standing (mmHg)	137.63 ± 15.86	132.18 ± 17.8	0.020
DBP standing (mmHg)	87.08 ± 11.25	84.35 ± 9.76	0.129
HR standing (bpm)	83.18 ± 10.86	81.15 ± 11.13	0.192
Deep breathing	23.38 ± 20.35	23.2 ± 18.18	0.948
Valsalva ratio	3.63 ± 3.06	3 ± 2.01	0.198
Schellong test (mmHg)	5.38 ± 11.38	5.88 ± 14.07	0.863
Handgrip	10.53 ± 10.82	10.15 ± 7.91	0.828
Right radius tuning fork	5.88 ± 1.38	6.15 ± 0.89	0.169
Left radius tuning fork	5.95 ± 1.18	6.18 ± 0.96	0.202
Right hallux tuning fork	5.03 ± 1.4	5.05 ± 1.48	0.915
Left hallux tuning fork	4.95 ± 1.45	5.1 ± 1.57	0.520
NTSS	2.74 ± 2.67	3.07 ± 3	0.470
Metabolic parameters			
Glucose (mmol/L)	9.43 ± 2.77	8.49 ± 2.14	0.038
Insulin (pmol/L)	209.82 ± 174.59	168.95 ± 138.23	0.100
HOMA_IR_	12.72 ± 9.23	9.55 ± 7.11	0.099
Fructosamine (µmol/L)	309.18 ± 42.07	297.45 ± 36.14	0.001
Triglyceride (mmol/L)	1.96 ± 0.79	1.95 ± 0.98	0.961
Kidney function			
Creatinine (µmol/L)	81 ± 21.8	78.48 ± 21.62	0.123
eGFR (ml/min/1.73 m^2^)	80.88 ± 18.21	83.03 ± 19.56	0.141
MAU (mg/L)	56.73 ± 126.92	12.94 ± 23.01	0.032
ACR (mg/mmol)	4.37 ± 9.28	2.13 ± 4.2	0.082
Na (mmol/L)	139.43 ± 2.29	139.98 ± 2.13	0.094
K (mmol/L)	4.37 ± 0.38	4.48 ± 0.4	0.005
Inflammatory and hematological parameters			
hsCRP (mg/L)	5.15 ± 6.23	5.21 ± 8.17	0.947
We (mm/h)	11.88 ± 8.67	11.64 ± 9.36	0.686
RBC (T/L)	4.8 ± 0.47	4.87 ± 0.49	0.020
Hgb (g/L)	142 ± 14.1	144.23 ± 14.2	0.029
Htc (%)	40.9 ± 3.93	41.95 ± 3.95	0.002
MCV (fl)	85.46 ± 3.37	86.17 ± 3.12	0.014
MCH (pg)	29.61 ± 1.53	29.63 ± 1.28	0.833
PLT (G/L)	263.65 ± 55.45	275.2 ± 56.84	0.022
Hydroxyl free radical parameters			
Serum p-Tyr (µmol/L)	53.09 ± 13.35	56.7 ± 32.33	0.502
Serum m-Tyr (nmol/L)	45.83 ± 37.23	45.04 ± 34.4	0.905
Serum o-Tyr (nmol/L)	52.1 ± 36.54	54.44 ± 30.76	0.760
Serum Phe (µmol/L)	43.26 ± 10.08	48.87 ± 23.02	0.144
Serum p-Tyr/Phe (µmol/µmol)	1.29 ± 0.41	1.18 ± 0.27	0.144
Serum m-Tyr/Phe (nmol/µmol)	1.23 ± 1.39	1.01 ± 0.81	0.316
Serum o-Tyr/Phe (nmol/µmol)	1.31 ± 1.06	1.24 ± 0.84	0.711
Serum m-Tyr/p-Tyr (nmol/µmol)	0.88 ± 0.7	0.93 ± 0.81	0.699
Serum o-Tyr/p-Tyr (nmol/µmol)	1.06 ± 0.78	1.1 ± 0.79	0.795
Serum (m-Tyr+o-Tyr)/Phe (nmol/µmol)	2.55 ± 2.04	2.24 ± 1.27	0.409
Serum (m-Tyr+o-Tyr)/p-Tyr (nmol/µmol)	1.93 ± 1.07	2.03 ± 1.34	0.698
Urinary p-Tyr (µmol/L)	63.27 ± 59.32	52.56 ± 40.42	0.103
Urinary m-Tyr (nmol/L)	3.44 ± 319.36	275.99 ± 415.87	0.781
Urinary o-Tyr (nmol/L)	5562.68 ± 15,755.06	1394.15 ± 1396.59	0.100
Urinary Phe (µmol/L)	56.96 ± 58.28	48.01 ± 31.42	0.331
Urinary p-Tyr/Phe (µmol/µmol)	1.88 ± 2.89	1.53 ± 1.81	0.530
Urinary m-Tyr/Phe (nmol/µmol)	10.78 ± 20.44	7.75 ± 11.12	0.424
Urinary o-Tyr/Phe (nmol/µmol)	96.56 ± 141.96	66.94 ± 213.28	0.408
Urinary m-Tyr/p-Tyr (nmol/µmol)	7.5 ± 7.64	7.8 ± 11.76	0.901
Urinary o-Tyr/p-Tyr (nmol/µmol)	77.28 ± 109.45	34.47 ± 36.24	0.020
Urinary (m-Tyr+o-Tyr)/Phe (nmol/µmol)	107.34 ± 146.24	74.7 ± 214.22	0.373
Urinary (m-Tyr+o-Tyr)/p-Tyr (nmol/µmol)	84.79 ± 109.55	42.26 ± 39.71	0.024
Urinary p-Tyr/creatinine (µmol/mmol)	38.78 ± 157.63	8.75 ± 5.08	0.243
Urinary m-Tyr/creatinine (nmol/mmol)	557.46 ± 2307.09	53.55 ± 68.91	0.183
Urinary o-Tyr/creatinine (nmol/mmol)	1279.49 ± 3653.87	250.1 ± 235.08	0.089

Data are presented as mean ± SD; significance was tested using a paired sample *t*-test. Abbreviations: ACR, urinary albumin per creatinine ratio; BMI, body mass index; DBP, diastolic blood pressure; eGFR, estimated glomerular filtration rate; Hgb, hemoglobin; HOMA_IR_, Homeostasis Model Assessment of Insulin Resistance; HR, heart rate; hsCRP, high-sensitive C-reactive protein; Htc, hematocrit; MAU, microalbuminuria; MCH, mean corpuscular hemoglobin concentration; MCV, mean corpuscular volume; m-Tyr, meta-tyrosine; NTSS, Neuropathy Total Symptom Score; o-Tyr, ortho-tyrosine; Phe, phenylalanine; PLT, platelet number; p-Tyr, para-tyrosine; RBC, red blood cell count; SBP, systolic blood pressure; We, blood sedimentation rate.

**Table 2:** Current perception threshold (CPT) results at baseline (Week 0) and after 4 weeks of SGLT-2 inhibitor therapy in the complete study population.

CPT Measurement	Week 0 (CPT)	Week 4 (CPT)	*p*
2000 Hz peroneal nerve left	376.2 ± 132.96	399.58 ± 153.44	0.416
2000 Hz peroneal nerve right	430.18 ± 150.37	403.7 ± 134.01	0.367
2000 Hz median nerve left	280.6 ± 130.81	284.08 ± 127.4	0.867
2000 Hz median nerve right	289 ± 115.87	298 ± 107.4	0.565
250 Hz peroneal nerve left	181.08 ± 96.89	178.28 ± 89.46	0.828
250 Hz peroneal nerve right	198.18 ± 103.82	178.73 ± 72.38	0.270
250 Hz median nerve left	101.38 ± 49.43	93.63 ± 56.21	0.458
250 Hz median nerve right	103.2 ± 54.13	112.65 ± 75.16	0.450
5 Hz peroneal nerve left	102.95 ± 62.92	110.79 ± 61.51	0.219
5 Hz peroneal nerve right	123.33 ± 57.54	126.55 ± 73.7	0.787
5 Hz median nerve left	76.08 ± 53.79	75.5 ± 96.63	0.973
5 Hz median nerve right	60.98 ± 36.88	75.78 ± 58.16	0.087

The table shows mean ± SD CPT (10 μA) for each nerve and frequency, along with *p*-values from paired-sample *t*-tests comparing Week 0 vs. Week 4. Abbreviation: CPT, current perception threshold.

**Table 3:** Predictors of peripheral neuropathy, red blood cell, and albuminuria parameters at baseline (Week 0) before SGLT-2 inhibitor therapy.

Dependent	Predictor	B	CI (95%)		*p*
NP2000 Hz	Um-Tyr	−0.142	−0.237	−0.047	0.004
	So-Tyr	1.123	0.271	1.974	0.011
NM2000 Hz	Um-Tyr/p-Tyr	3.932	0.524	7.340	0.025
NP250 Hz	Um-Tyr/p-Tyr	4.800	1.054	8.547	0.013
NM250 Hz	So-Tyr	−0.422	−0.753	−0.090	0.014
NP5 Hz	BMI	3.575	0.080	7.070	0.045
NM5 Hz	-				
Hgb	S(m-Tyr+o-Tyr)/p-Tyr	−10.166	−13.280	−7.052	<0.001
	BMI	0.829	0.122	1.536	0.023
Htc	S(m-Tyr+o-Tyr)/p-Tyr	−2.546	−3.362	−1.730	<0.001
	Um-Tyr/creatinine	0.000	0.000	0.001	0.034
MCV	Um-Tyr	0.004	0.001	0.006	0.003
	SBP (standing)	0.077	0.027	0.127	0.004
	fructosamine	0.024	0.002	0.045	0.032
MAU	U(m-Tyr+o-Tyr)/Phe	−0.738	−0.996	−0.481	<0.001
	U(m-Tyr+o-Tyr)/p-Tyr	1.109	0.843	1.374	<0.001

Data were obtained using a linear regression (stepwise method) model with the following independent parameters: BMI, SBP (standing), fructosamine, serum m-Tyr, serum o-Tyr, serum m-Tyr/Phe, serum o-Tyr/Phe, serum m-Tyr/p-Tyr, serum o-Tyr/p-Tyr, serum (m-Tyr+o-Tyr)/Phe, serum (m-Tyr+o-Tyr)/p-Tyr, urinary m-Tyr, urinary o-Tyr, urinary m-Tyr/Phe, urinary o-Tyr/Phe, urinary m-Tyr/p-Tyr, urinary o-Tyr/p-Tyr, urinary (m-Tyr+o-Tyr)/Phe, urinary (m-Tyr+o-Tyr)/p-Tyr, urinary m-Tyr/creatinine, and urinary o-Tyr/creatinine (evaluated at both the actual week and delta).

## Data Availability

The original contributions presented in this study are included in the article. Further inquiries can be directed to the corresponding author.

## Supplementary Materials

The following supporting information can be downloaded at: https://www.mdpi.com/article/10.3390/antiox14030289/s1, Figure S1: Correlation between platelet count (PLT) and the (m-Tyr+o-Tyr)/p-Tyr ratio in serum (panels A, B) and urine (panels C, D) at week 0 (A, C) and week 4 (B, D) of SGLT-2 inhibitor therapy; Figure S2: Correlation between microalbuminuria (MAU) and the (m-Tyr+o-Tyr)/p-Tyr ratio in serum (panels A, B) and urine (panels C, D) at week 0 (A, C) and week 4 (B, D) of SGLT-2 inhibitor therapy.

## Abbreviations

The following abbreviations are used in this manuscript:

ACR	Albumin-to-creatinine ratio
ALA	Alpha-lipoic acid
ALP	Alkaline phosphatase
ALT	Alanine aminotransferase
AST	Aspartate aminotransferase
BMI	Body mass index
bpm	Beats per minute
CPT	Current perception threshold
CRP	C-reactive protein
DBP	Diastolic blood pressure
DKA	Diabetic Ketoacidosis
DN4	Douleur Neuropathique en 4 (neuropathic pain questionnaire)
DSPN	Distal symmetric polyneuropathy
eGFR	Estimated glomerular filtration rate
ESR	Erythrocyte sedimentation rate (also shown as “We” in some tables)
GGT	Gamma-glutamyl transferase
HbA1c	Hemoglobin A1c
Hgb	Hemoglobin
HOMA_IR_	Homeostasis Model Assessment of Insulin Resistance
HPLC	High-Performance Liquid Chromatography
HR	Heart rate
Htc	Hematocrit
INR	International normalized ratio
LDH	Lactate dehydrogenase
MCH	Mean corpuscular hemoglobin
MCV	Mean corpuscular volume
m-Tyr	Meta-tyrosine
MAU	Microalbuminuria
NCS	Nerve conduction study
NDS	Neuropathy Disability Score
NTSS	Neuropathy Total Symptom Score
o-Tyr	Ortho-tyrosine
p-Tyr	Para-tyrosine
Phe	Phenylalanine
PLT	Platelet count
RBC	Red blood cell
RR	R–R interval (time between R-waves on ECG)
SBP	Systolic blood pressure
SGLT-2	Sodium–glucose cotransporter-2
SPSS	Statistical Package for the Social Sciences
T2D	Type 2 diabetes
TCA	Trichloroacetic acid
