# Supplementary material for: The Impact of SGLT-2 Inhibitors on Hydroxyl Radical Markers and Diabetic Neuropathy: A Short-Term Clinical Study
Source: Antioxidants (Basel). 2025 Feb 28;14(3):289. doi: 10.3390/antiox14030289 (PMC11939660; doi:10.3390/antiox14030289)
Supplement: Supplementary file 1 [file antioxidants-14-00289-s001.zip › antioxidants-3450944-supplementary.pdf]

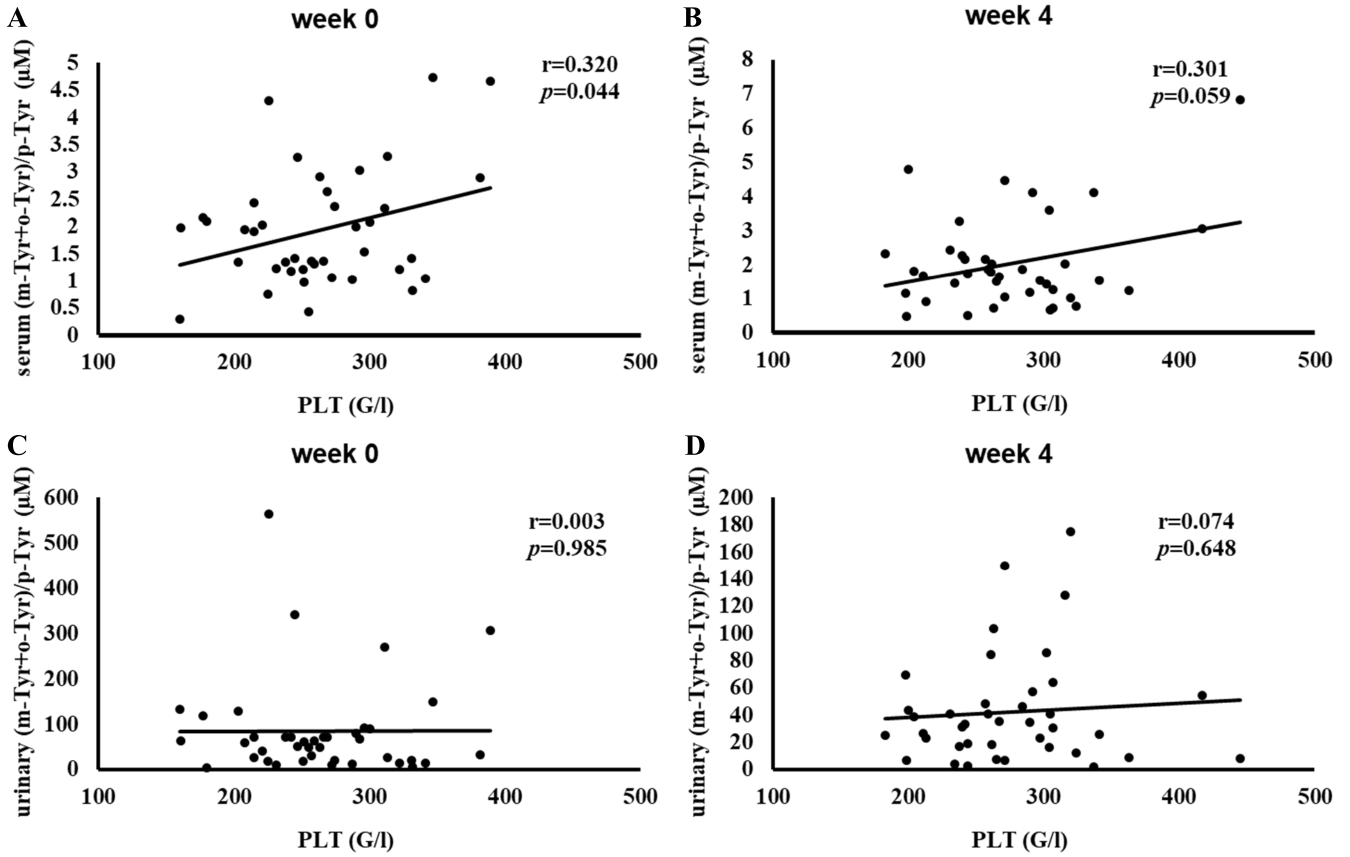

Figure S1. Correlation between platelet count (PLT) and the (m-Tyr + o-Tyr)/p-Tyr ratio in serum (panels **A**, **B**) and urine (panels **C**, **D**) at week 0 (**A**, **C**) and week 4 (**B**, **D**) of SGLT-2 inhibitor therapy. Each panel indicates the Pearson correlation coefficient (*r*) and corresponding *p*-value. The fitted line represents the linear regression trend.
Abbreviations**:** PLT, platelet count; m-Tyr, meta-tyrosine; o-Tyr, ortho-tyrosine; p-Tyr, para-tyrosine.

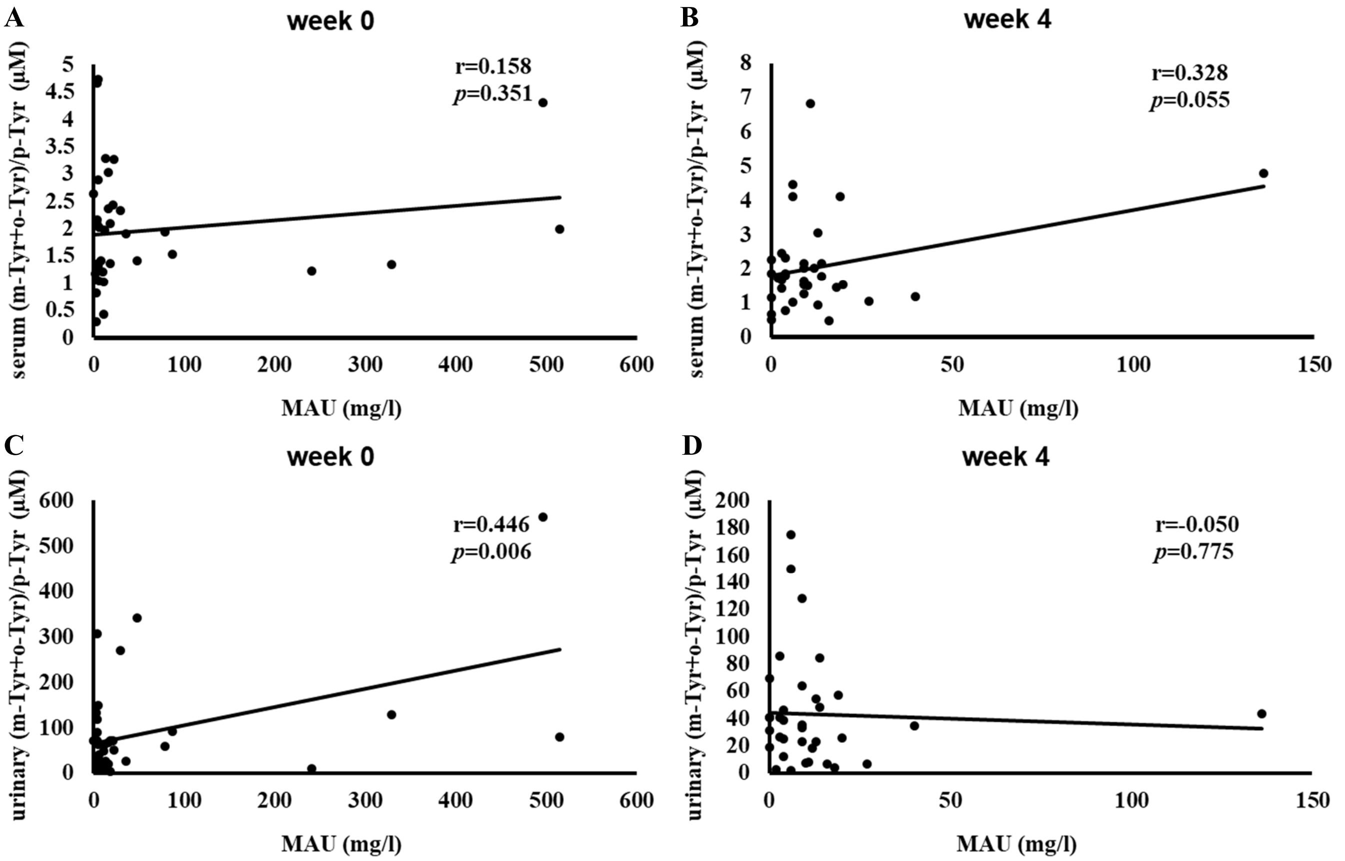

Figure S2. Correlation between microalbuminuria (MAU) and the (m-Tyr + o-Tyr)/p-Tyr ratio in serum (panels **A**, **B**) and urine (panels **C**, **D**) at week 0 (**A**, **C**) and week 4 (**B**, **D**) of SGLT-2 inhibitor therapy. Each panel indicates the Pearson correlation coefficient (*r*) and corresponding *p*-value. The fitted line represents the linear regression trend. Abbreviations**:** MAU, microalbuminuria; m-Tyr, meta-tyrosine; o-Tyr, ortho-tyrosine; p-Tyr, para-tyrosine.
